# Pink Bollworm Resistance to Bt Toxin Cry1Ac Associated with an Insertion in Cadherin Exon 20

**DOI:** 10.3390/toxins11040186

**Published:** 2019-03-28

**Authors:** Ling Wang, Yuemin Ma, Xueqin Guo, Peng Wan, Kaiyu Liu, Shengbo Cong, Jintao Wang, Dong Xu, Yutao Xiao, Xianchun Li, Bruce E. Tabashnik, Kongming Wu

**Affiliations:** 1 Key Laboratory of Integrated Pest Management on Crops in Central China, Ministry of Agriculture, Hubei Key Laboratory of Crop Disease, Insect Pests and Weeds Control, Institute of Plant Protection and Soil Fertility, Hubei Academy of Agricultural Sciences, Wuhan 430064, China; wanglin20504@163.com (L.W.); wanpenghb@126.com (P.W.); congshengbo@163.com (S.C.); wjt217@126.com (J.W.); ztb799@163.com (D.X.); 2 State Key Laboratory for Biology of Plant Diseases and Insect Pests, Institute of Plant Protection, Chinese Academy of Agricultural Sciences, Beijing 100193, China; 3 School of Life Science, Central China Normal University, Wuhan 430079, China; ymma@mails.ccnu.edu.cn (Y.M.); 15207168391@163.com (X.G.); liukaiyu@mail.ccnu.edu.cn (K.L.); 4 Agricultural Genomics Institute at Shenzhen, Chinese Academy of Agricultural Sciences, Shenzhen 518120, China; xiao20020757@163.com; 5 Department of Entomology, University of Arizona, Tucson, AZ 85721, USA; lxc@email.arizona.edu (X.L.); brucet@cals.arizona.edu (B.E.T.)

**Keywords:** Bt cotton, resistance mechanism, Cry1Ac, *Bacillus thuringiensis*, *Pectinophora gossypiella*, genetically engineered crop, transposon

## Abstract

Insecticidal proteins from *Bacillus thuringiensis* (Bt) are widely used to control insect pests, but their efficacy is reduced when pests evolve resistance. We report on a novel allele (*r16*) of the cadherin gene (*PgCad1*) in pink bollworm (*Pectinophora gossypiella*) associated with resistance to Bt toxin Cry1Ac, which is produced by transgenic cotton. The *r16* allele isolated from a field population in China has 1545 base pairs of a degenerate transposon inserted in exon 20 of *PgCad1*, which generates a mis-spliced transcript containing a premature stop codon. A strain homozygous for *r16* had 300-fold resistance to Cry1Ac, 2.6-fold cross-resistance to Cry2Ab, and completed its life cycle on transgenic Bt cotton producing Cry1Ac. Inheritance of Cry1Ac resistance was recessive and tightly linked with *r16*. Compared with transfected insect cells expressing wild-type *PgCad1*, cells expressing *r16* were less susceptible to Cry1Ac. Recombinant cadherin protein was transported to the cell membrane in cells transfected with the wild-type *PgCad1* allele, but not in cells transfected with *r16*. Cadherin occurred on brush border membrane vesicles (BBMVs) in the midgut of susceptible larvae, but not resistant larvae. These results imply that the *r16* allele mediates Cry1Ac resistance in pink bollworm by interfering with the localization of cadherin.

## 1. Introduction

Genetically engineered crops producing insecticidal proteins from *Bacillus thuringiensis* (*Bt*) were cultivated on a cumulative total of over 930 million hectares worldwide from 1996 to 2017 [1]. These Bt crops kill some key pests while decreasing reliance on conventional insecticide treatments and reducing harm to non-target species including arthropod natural enemies and vertebrates [2,3,4,5]. However, these benefits are diminished when pests evolve resistance [6,7,8,9]. Some populations of seven major pests have evolved practical resistance to Bt crops, which is defined as field-evolved resistance that reduces the efficacy of Bt crops and has practical consequences for pest management [8]. 

Binding of widely used crystalline (Cry) toxins from Bt to larval midgut receptor proteins is essential for toxicity, and disruption of this binding is the most common mechanism of insect resistance to Cry toxins [10,11,12,13]. Insect resistance to Cry toxins is often associated with mutations affecting one of four types of midgut receptor proteins: cadherin, ATP-binding cassette (ABC) transporters, aminopeptidase N, and alkaline phosphatase [10,11,12,13].

Here, we focus on a novel cadherin allele associated with resistance to Bt toxin Cry1Ac in pink bollworm (*Pectinophora gossypiella*), a global pest of cotton [14]. Transgenic cotton producing Cry1Ac was highly effective against pink bollworm when first introduced. In India, however, the practical resistance of pink bollworm to Cry1Ac evolved rapidly [15,16]. In the United States and China, pink bollworm has not evolved practical resistance to Cry1Ac in the field [17,18], but laboratory selection of progeny derived from field-collected insects from those two countries has yielded many strains resistant to Cry1Ac [19,20,21,22,23,24], indicating the potential for field-evolved resistance. Therefore, understanding the genetic basis and mechanism of pink bollworm resistance to Cry1Ac may be useful for developing and implementing resistance management strategies. Previous work has identified 15 mutant *PgCad1* alleles (*r1*–*r15*) associated with pink bollworm resistance to Cry1Ac [19,20,21,22,23,24,25]. Here we identified and analyzed a novel *PgCad1* resistance allele (*r16*) in pink bollworm from the Yangtze River Valley of China. 

## 2. Results

### 2.1. Isolation and Characterization of the Novel PgCad1 Allele r16

The AQ65 strain originated from a single pair mating between a male (#65) collected in 2013 from Anqing in the Yangtze River Valley and a female from the lab-selected Cry1Ac-resistant strain AZP-R from Arizona [20]. Survival of the F_1_ larvae fed on a diet with a concentration of 10 μg Cry1Ac per mL diet was 40% (n = 72). PCR revealed that all of the F_1_ larvae surviving exposure to Cry1Ac had one copy of the *r1* cadherin allele from AZP-R and the male parent did not have *r1*, and the female parent was an *r1r1* homozygote. 

Resistance to Cry1Ac in previously studied strains of pink bollworm is associated with recessive mutations affecting cadherin [19,22,23,24] and the results described above suggested that male #65 carried a recessive cadherin resistance allele other than *r1*. We used a series of single-pair matings, DNA screening, and selection with Cry1Ac to eliminate the *r1* allele and obtain a strain (AQ65) harboring a novel cadherin resistance allele (Figure S1), which we named *r16*, following the nomenclature for *PgCad1* resistance alleles [20,23,24,25].

**Figure 1:**
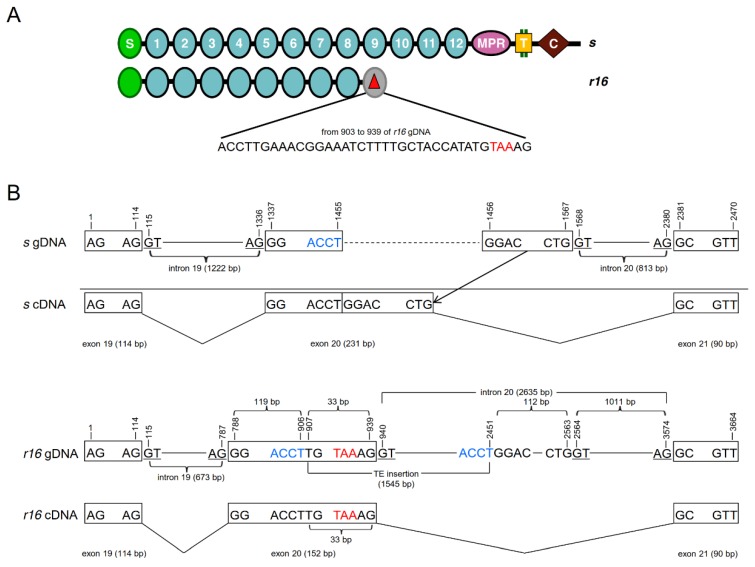
*PgCad1 r16* mutation. (**A**) *r16* and *s* allele protein alignment showing amino-terminal membrane signal sequence (S), cadherin repeats (1–12), membrane proximal region (MPR), transmembrane region (T), and cytoplasmic domain (C). The red triangle indicates the truncation of the protein predicted from *r16* because of the premature stop codon (red letters TAA). (**B**) *r16* and *s* allele gDNA/cDNA alignment. The red letters TAA indicate the premature stop codon and the blue letters ACCT indicate the target site duplications (TSDs). Underlined letters GT/AG indicate a splicing site.

Sequencing the full-length cDNA from AQ65 revealed that *r16* has various deletions and a premature stop codon at the 3’ end of a deletion (base pairs (bp) 3220 to 3222; Figure S2). As a result of the premature stop codon, *r16* cDNA encodes a truncated cadherin protein missing 662 amino acids including cadherin repeat 9 to 12 (CR9-12), the membrane proximal region (MPR), transmembrane domain (TM), and cytoplasmic domain (CYT) (Figure 1A and Figure S3). 

To determine what caused the deletions and introduction of the premature stop codon (Figure S2), we amplified the DNA flanking the premature stop codon (Figure 1B, Table S1). The length of the gDNA was 3664 bp from *r16* in AQ65 and 2470 bp from the corresponding wild-type (*s*) allele in the susceptible APHIS-S strain (Table S1). Alignment of the gDNA and cDNA of *r16* and *s* alleles in this region reveals the insertion of 1545 bp into exon 20 at bp 906 (Figure 1B and Figure S4). In contrast, only one single nucleotide polymorphism (SNP) between the two alleles occurred in exon 19 and another in exon 21 (Figure 1B and Figure S4). In the aligned regions of introns 19 and 20, the *r16* and *s* alleles share only 83% and 92% sequence identity, respectively (Figure S4). PCR of cadherin gDNA from male #65 using allele-specific primers (Figure S5) confirmed his genotype was *r16s.*

The 1545-bp insertion in exon 20 of *r16* appears to be a degenerate transposon as it is flanked by two ACCT target site duplications (TSDs) (Figure 1B and Figure S4). While we found no ORF or other recognizable features in this insertion, the sequence similarity from bp 660 to 719 (i.e., 1566 to 1625 of *r16* gDNA) with the Penelope non-LTR transposon T2_ suggests that it is probably a degenerate non-LTR transposon. 

This transposon contains a 5’ GT splicing site at bp 940 (33 bp downstream of its insertion site at bp 907), and a premature stop codon TAA just 2 bp upstream of this 5’ GT splicing site (Figure 1B and Figure S4). Consequently, insertion of this transposon into exon 20 causes mis-splicing of intron 20 through the use of the aforementioned transposon 5’ GT splicing site at bp 940 rather than its original 5’ GT splicing site at bp 2564 for intron 20 (Figure 1B and Figure S4). This leads to the formation of *r16* mRNA, whose exon 20 contains the 5’ 119 bp (from bp 788 to 906 in Figure 1B) of the *s* allele exon 20 and the 5’ 33 bp (with a premature stop codon; from bp 907 to 939 in Figure 1B) of the transposon, but skips the 3’ 112 bp (from bp 2452 to 2563 in Figure 1B) of the *s* allele exon 20. In contrast, the other indels in intron 19 and 20 (Figure S4) do not contribute to *r16* cDNA.

### 2.2. Inheritance of Cry1Ac Resistance and Cross-Resistance to Cry2Ab

The concentration (in µg Cry1Ac per mL diet) that killed 50% of larvae (LC50) was 29.5 for AQ65 versus 0.097 for APHIS-S, yielding a resistance ratio of 300 for AQ65 (Table 1). The resistance ratios of the F_1_ offspring from reciprocal crosses between AQ65 and APHIS-S were 5.8 and 4.7 (Table 1). No significant difference in LC_50_ occurred between the progeny from the two reciprocal crosses, showing that the resistance of AQ65 is autosomal, with no evidence of sex linkage or maternal effects (Table 1). For larvae fed on a diet with 10 μg Cry1Ac per mL diet, survival was 80% for AQ65 and 0% for APHIS-S, and 0% for the F_1_ offspring from both reciprocal crosses between AQ65 and APHIS-S (n = 72 to 96 larvae for each strain and each hybrid cross). This produced a value of 0 for the dominance parameter *h*, indicating a completely recessive inheritance of resistance at this concentration.

The LC_50_ of Cry2Ab (in µg Cry2Ab per ml diet) was 0.157 for APHIS-S and 0.408 for AQ65, yielding a resistance ratio of 2.6 (Table S2). The LC_50_ of Cry2Ab was significantly higher for AQ65 than APHIS-S by the conservative criterion of non-overlap of their 95% fiducial limits (Table S2). Thus, these results indicate weak, but statistically significant cross-resistance of AQ65 to Cry2Ab (Table S2).

### 2.3. Genetic Linkage between r16 Allele and Cry1Ac Resistance 

We used genetic linkage analysis to test the hypothesis that resistance to Cry1Ac in AQ65 is linked with *r16*. Five backcross families were produced by five single-pair crosses, each between an AQ65 female and an F_1_ male (AQ65 × APHIS-S). In 147 larvae from the five backcross families that survived on a control diet, the percentage of *r16r16* homozygotes was 48%, which did not differ from the expected 50% (t-test, df = 4, t = −0.79, *P* = 0.47). In contrast, 100% of the 103 larvae that survived on a diet with 10 μg Cry1Ac per mL diet were *r16r16*, and the proportion of *r16r16* survivors was significantly higher on the treated diet than the control diet (Fisher’s exact test, *P* < 0.001) (Table S3). These results indicate tight genetic linage between *r16* and resistance to Cry1Ac.

### 2.4. Life History Traits of AQ65 on Bt and Non-Bt Cotton

Larval survival on Bt cotton bolls was significantly higher for AQ65 (19.4%) than APHIS-S (0.0%) (t-test, t = 12.1, df = 4, *P* < 0.0001, Table S4). On non-Bt cotton bolls, larval survival did not differ significantly between AQ65 (38.3%) and APHIS-S (31.1%) (t-test, t = 2.5, df = 4, *P* = 0.069, Table S4), the developmental time from neonate to pupa was significantly longer for AQ65 (17.1 days) than APHIS-S (15.0 days) (Table 2), and pupal weight did not differ significantly between the two strains (Table 2). These results indicate a fitness cost associated with resistance in AQ65 affected development time, but not larval survival or pupal weight.

For AQ65, survival from neonate to adult and the proportion of female adults were significantly lower on Bt cotton than non-Bt cotton (Fisher’s exact test, *P* < 0.00001 for each trait). In contrast, no significant difference occurred between AQ65 females from Bt versus non-Bt cotton bolls in the number of eggs laid per female (t = 1.3, df = 8, *P* = 0.23) or the percentage of their eggs hatching (t = 0.77, df = 16, *P* = 0.45) (Table 3). Based on the life history traits summarized above, the net reproductive rate for AQ65 was 3.5 times higher on non-Bt cotton than Bt cotton, indicating incomplete resistance of AQ65 to Bt cotton.

### 2.5. Transfected Cells: PgCad1 Localization and Susceptibility to Cry1Ac

**Figure 2:**
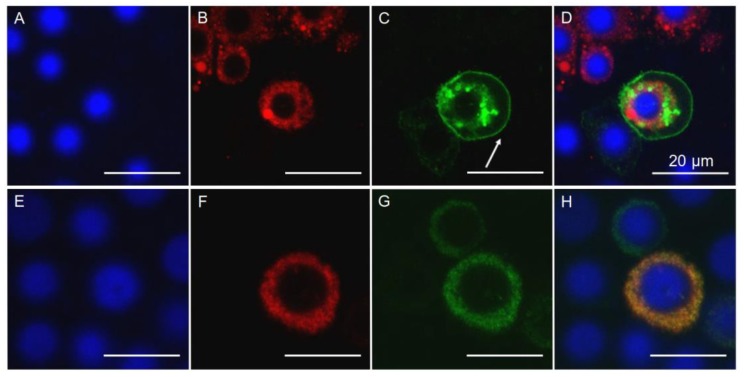
Cellular localization of PgCad1 proteins within Hi5 cells. Hi5 cells transfected with pIE2-sPgCad1-GFP (**A**–**D**) and pIE2-r16PgCad1-GFP (**E**–**H**). Nuclei stained with Hoechst 3342 are shown in blue, dsRED-labeled endoplasmic reticulum is shown in red, and GFP-labeled PgCad1 fusion proteins are shown in green. Superimposed images from (**A**–**C**) are shown in (**D**) and from (**E**–**G**) in (**H**). The arrow in (**C**) indicates the cell membrane. Bar = 20 μm.

We transfected the recombinant expression vector containing an *s* or *r16* allele into Hi5 cells to produce the PgCad1-GFP fusion protein sPgCad1-GFP or r16PgCad1-GFP (Figure 2). Transfection efficiencies (mean % ± SE) did not differ significantly between sPgCad1-GFP (69 ± 9%) and r16PgCad1-GFP (64 ± 5%) (t-test, t = 0.43, df = 4, *P* = 0.69). Immunoblots demonstrated that molecular weights of the recombinant PgCad1-GFP fusion proteins produced by transformed Hi5 cells were as expected (sPgCad1-GFP = 253 kDa and r16PgCad1-GFP = 168 kDa) (Figure S6). Hi5 cells producing PgCad1-GFP fusion protein were also transformed with the pIE2-DsRed2-ER vector that expressed an endoplasmic reticulum (ER) tag protein and generated red fluorescence (Figure 2). The fusion protein sPgCad1-GFP appeared primarily with the cell membrane (Figure 2A–D), while r16PgCad1-GFP occurred with the ER (Figure 2E–H). 

**Figure 3:**
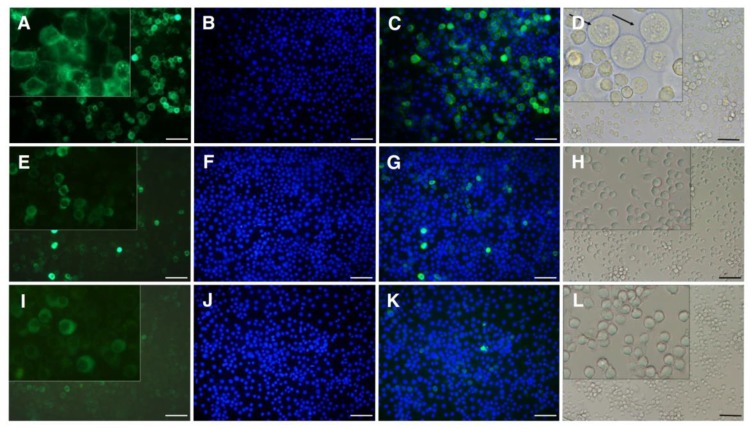
Susceptibility to Cry1Ac of Hi5 cells producing PgCad1 proteins. Hi5 cells transfected with pIE2-sPgCad1-GFP (**A**–**D**), pIE2-r16PgCad1-GFP (**E**–**H**) or the empty vector pIE2-GFP (**I**–**L**) were treated with Cry1Ac (10 μg Cry1Ac per ml for cells producing sPgCad1-GFP and 40 μg Cry1Ac per ml for r16PgCad1-GFP and GFP cells) and observed for swelling using fluorescence microscopy. Nuclei stained with Hoechst 3342 are shown in blue and PgCad1-GFP fusion proteins are shown in green. Superimposed images from (**A**–**B**) are shown in (**C**), from (**E**–**F**) in (**G**), and from (**I**–**J**) in (**K**). Arrows in (**D**) indicate representative swollen cells. Bars shown in (**D**, **H**, and **L**) = 200 μm.

When cells were treated with Cry1Ac, swelling and cell lysis occurred in the cells expressing sPgCad1-GFP (Figure 3A–D), but not in the cells expressing r16PgCad1-GFP (Figure 3E–H) or GFP (Figure 3I–L). For cells expressing sPgCad1-GFP, the concentration of Cry1Ac causing swelling in half of the cells (EC50) was 7.3 μg per mL (95% confidence interval = 6.2 to 8.4). In contrast, no swelling occurred in the cells expressing r16PgCad1-GFP treated with the highest toxin concentration tested (40 μg Cy1Ac per mL).

### 2.6. Localization of PgCad1 in APHIS-S and AQ65

**Figure 4:**
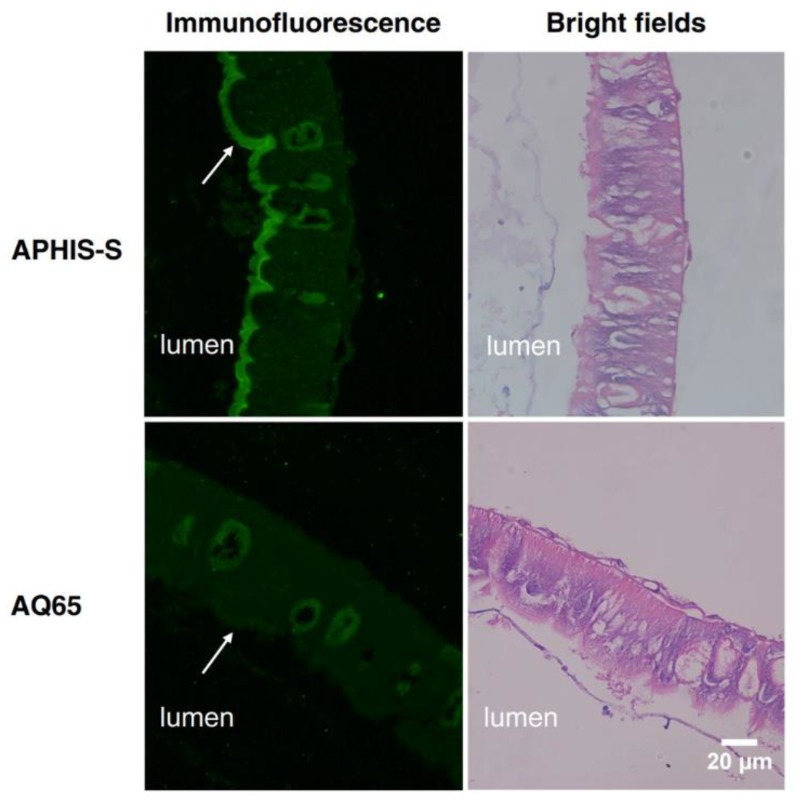
Localization of the cadherin protein in midgut tissue sections of the fourth instar APHIS-S and AQ65 larvae. The cadherin protein was revealed by immunofluorescence using rabbit anti-PgCad1 antibody (see Methods Section for details). Arrows point to the brush border microvilli membrane. Bar, 20 µm.

We analyzed the localization of PgCad1 in midgut tissue sections of larvae from APHIS-S and AQ65 by immunofluorescence detection using the anti-PgCad1 antibody. The PgCad1 protein was mainly located on the brush border membrane vesicles (BBMVs) for APHIS-S larvae, but not for AQ65 (Figure 4). 

## 3. Discussion

In this study, we identified and analyzed a novel cadherin allele (*r16*) of pink bollworm associated with resistance to Bt toxin Cry1Ac. The *r16* allele cDNA has a premature stop codon (Figure S2) resulting from the insertion of 1545 bp in exon 20 (Figure 1B and Figure S4). The 1545 bp insertion appears to be a degenerate non-LTR transposon based on its similarity with the Penelope non-LTR transposon T2. This insertion causes mis-splicing of exon 20 and introduces a premature stop codon (Figure 1B and Figure S4). The presence of many indels, the low sequence similarity in introns 19 and 20 between *s* and *r16* allele gDNA as well as the degeneration of the transposon, suggest that this transposon jumped into exon 20 long before the use of Bt cotton. 

The premature stop codon at bp 3220 to 3222 represents a new mutation that distinguishes *r16* from the previously reported *r1*–*r15* cadherin resistance alleles [19,20,23,25]. The mechanism of resistance to Cry1Ac, which entails the failure of cadherin to localize on the cell membrane, is similar for *r16* and *r13* from the Yangtze River Valley [24]. For pink bollworm, a total of 23 different transcripts have been identified from the 15 previously reported cadherin alleles, of which 16 transcripts have premature stop codons [19,20,23,25]. In terms of the location of the disruptive mutation, the *r16* allele is most similar to the *r2* allele; both have a premature stop codon expected to yield a cadherin protein that is missing a portion of the CR domain as well as downstream domains [20]. Moreover, the GYBT and LF60 strains of *Helicoverpa armigera* had high resistance to Cry1Ac caused by the premature termination of cadherin and ABCC2 proteins, respectively [27,28]. These results indicate that premature termination of protein translation is a common phenomenon in alleles conferring resistance to Cry1Ac. 

In this study, 19.4% of larvae from AQ65 survived on Bt cotton (Table S4), and *r16* was completely recessive at the toxin concentration of 10 µg Cry1Ac per mL diet. In previous work, the pink bollworm cadherin resistance alleles *r1*, *r2*, *r3,* and *r13* were recessive and enabled survival on Bt cotton [20,24,29], whereas *r4* was recessive, but did not confer survival on Bt cotton [22]. Most of the other previously identified cadherin alleles were detected in preserved larvae that survived on Bt cotton in the field in India, and their dominance was not evaluated [25]. 

Although Bt cotton producing Cry1Ac is still effective against pink bollworm in the Yangtze River Valley [18], the work described here brings the total of cadherin resistance alleles reported from that region to four (*r13*, *r14*, *r15*, *r16*), which signals the continuing risk of the evolution of resistance to Cry1Ac in pink bollworm. Thus, monitoring remains important for tracking the frequency of resistance in the field. Farmers in other countries such as Australia and the United States have switched from Cry1Ac-producing cotton to multi-toxin Bt cotton [8]. Given the low level of cross-resistance to Cry2Ab caused by resistance to Cry1Ac in pink bollworm seen here and previously [24,30,31], switching to dual-toxin Bt cotton that produces Cry1Ac and Cry2Ab could help to sustain the efficacy of Bt cotton against pink bollworm in China. 

## 4. Materials and Methods

### 4.1. Insects and Bt Toxins

We used three strains of pink bollworm: one susceptible strain (APHIS-S) and two resistant strains (AZP-R and AQ65). The APHIS-S strain originated from Arizona, in the United States, and was reared in the laboratory for >30 years without exposure to any pesticides or Bt toxins [24,32]. AZP-R originated from Arizona and has been selected repeatedly in the lab for resistance to Cry1Ac [19,33]. The AQ65 strain was started with a single pair mating between a male (#65) collected in October 2013 from a raw cotton purchasing station as a third generation larva from Anqing in Anhui Province of the Yangtze River Valley of China, and a female from AZP-R with cadherin genotype *r1r1*. The F_1_ offspring of that cross were screened with a 10 μg Cry1Ac per mL diet and survivors were collected to generate F_2_ offspring. After a series of single-pair crosses, DNA detection, and selection with Cry1Ac, we eliminated individuals carrying the *r1* allele and obtained the AQ65 strain with cadherin genotype *r16r16* (Figure S1).

All insects were maintained at 29 ± 1°C, relative humidity (RH) 50 ± 10% for larvae and 70 ± 10% for adults and a photoperiod of 16:8 (L:D). Larvae were reared on an artificial diet. To maintain resistance, larvae of AQ65 and AZP-R were fed on a diet with a 10 μg Cry1Ac per mL diet every fifth generation. We used the protoxin form of Cry1Ac and Cry2Ab, which were obtained as described previously [24].

### 4.2. Cloning and Sequencing of PgCad1

We used the fourth instar larvae of the AQ65 strain that survived on a diet containing 10 μg/mL Cry1Ac protoxin to clone and sequence the complete cDNA and partial gDNA of *PgCad1*. We isolated both total RNA and genomic DNA from the fourth instar individual (n = 8) using the RNA Extraction Kit (TaKaRa, Dalian, China). We obtained the first strand cDNA under the action of Reverse Transcriptase and full-length cDNA of *PgCad1* was cloned by PCR amplification as described previously [24]. To clone the gDNA flanking sequence of the *r16* mutation site, we used primers gF65 + gR65 (Table S1) and LA-Taq DNA polymerase (TaKaRa) for PCR amplification. PCR reaction conditions were as follows: the first step was 95°C for 2 min, and the second step was 32 cycles at 98°C for 10 s, 57°C for 30 s, and 72°C for 4 min, and the final step was incubation at 72°C for 10 min. PCR products were purified and cloned and sequenced as described before [24]. Alignments of gDNA and cDNA sequences of *r16* and *s* alleles were carried out by MUSCLE 3.8. We performed a BLAST search against the NCBI database and Censor search against the Repbase (http://www.girinst.org/repbase/index.html) to identify the insertion sequence in exon 20 of the *r16* allele. 

### 4.3. Bioassays

We used previously described diet incorporation bioassays [20,24,32] to determine the susceptibility of larvae from AQ65 and APHIS-S to the protoxin form of Cry1Ac and Cry2Ab [24], and from the F_1_ progeny of reciprocal crosses between AQ65 and APHIS-S to the protoxin form of Cry1Ac. We also performed boll bioassays to test the life history characteristics for larvae from AQ65 and a susceptible strain on both bolls obtained from Bt cotton expressing Cry1Ac (GuoXin H318) and bolls from non-Bt cotton (Simian-3). For the boll bioassays, three replicates were adopted with 11–16 bolls from Bt cotton and 14–15 bolls from non-Bt cotton per strain per replicate. A total of 1700 neonates were tested including 480 neonates from AQ65 and 350 neonates from APHIS-S on Bt bolls, and 440 neonates from AQ65 and 430 neonates from APHIS-S on non-Bt bolls. The life history traits such as the number of entry holes, exit holes, larval developmental days, pupal weight, pupal rate, emergence rate, sex ratio, eggs per female, and hatch rate were counted and recorded as previously described [24].

### 4.4. Inheritance of Resistance

Virgin males and females from the APHIS-S and AQ65 strain were used in reciprocal mass crosses. Twenty males from APHIS-S × twenty females from AQ65 and twenty females from APHIS-S × twenty males from AQ65 were placed into two different plastic boxes (2 L) to generate F_1_ progeny. Neonates of the two F_1_ progeny were used in the conducted diet bioassays according to the above Cry1Ac concentrations. We calculated the dominance parameter *h* as described previously [34]. Values of *h* ranged from 0 for completely recessive to 1 for completely dominant.

### 4.5. Genetic Linkage between r16 and Cry1Ac Resistance

To analyze the genetic linkage between Cry1Ac resistance and *r16*, we produced F_1_ offspring from a single-pair mating between a susceptible male and a resistant female from AQ65. We then produced five backcross families, and each of them was generated by a single-pair mating of a F_1_ male adult with a female adult from AQ65. As crossing over in Lepidoptera occurs only in males [35], we used F_1_ males rather than females to obtain backcross families and to test the genetic linkage between *r16* and Cry1Ac resistance. For each backcross family, roughly 100 newly hatched larvae were used for bioassays, of which about 40 were reared on a control diet and 60 were reared on a diet containing a 10 μg Cry1Ac per mL diet. We carried out DNA-based detection by specific PCR as above-mentioned to confirm the genotype of the fourth instar larvae that survived on the control diet or treated diet from each backcross family. A total of 250 larvae were tested to determine their genotypes including 147 larvae from untreated diet (n = 30, 30, 29, 30, and 28 larvae for each backcross family) and 103 larvae from a 10 μg Cry1Ac per mL diet (n = 20, 20, 21, 22, and 20 larvae for each family).

### 4.6. Expression Vectors and Transfection of Hi5 Cells

We isolated total RNA from both the AQ65 and APHIS-S strain as described above, amplified the intact open reading frames (ORFs) of *s* and *r16*, and then cloned the two ORFs individually into the expression vector pIE2-EGFP-N1 as described before [36] to generate recombinant vectors for expressing the fusion proteins sPgCad1-GFP and r16PgCad1-GFP. We transfected the recombinant vectors into the Hi5 cell line (T.ni BTI-Tn-5B1-4), which was provided by Prof. Peter Tijssen from the University of Quebec, Canada. The endoplasmic reticulum (ER) of insect Hi5 cells was marked by pDsRed2-ER as described previously [37]. We conducted cell transfection as described previously [24].

### 4.7. Expression of Fusion Protein PgCad1-GFP in Hi5 Cells

We transfected 2 μg of recombinant plasmids pIE2-sPgCad1-GFP, pIE2-r16PgCad1-GFP, or the empty plasmid pIE2-GFP (control group) into Hi5 cells. Cells were inoculated, lysed, and then analyzed by Western blot. We measured the total protein concentration of each group according to the BCA protein determination method [38] after cell lysis. The same amount of total protein for each sample (40 μg) was separated on a SDS-PAGE gel, then transferred onto a polyvinylidene difluoride membrane, followed by incubation with primary and secondary antibodies in proper order as previously described [24]. 

### 4.8. Toxicity Assays on Hi5 Cells

Cell toxicity assays were carried out 24 h after cell transfection. The transfected cells were treated with a series of concentrations of activated Cry1Ac (six treatments from 0 to 40 μg Cry1Ac per mL PBS) for one hour, then their morphology was observed under a fluorescence microscope. Cells toxicity was determined by the percentage of swollen cells as previously described [36]. Each treatment was replicated three times and the proportion of swollen cells for six visual fields of each repeat of each treatment were used to estimate cells toxicity.

### 4.9. Immunofluorescence Detection in Midgut Tissue Sections 

The midgut tissue was obtained by dissecting the fourth instar larvae from the susceptible and resistant strain, and then fixing with 4% paraformaldehyde. After dehydration, midgut tissue was embedded in paraffin and then cut into sections with a microtome as shown previously [39]. Midgut tissue sections were installed on silanized glass slides for dewaxing and rehydration as previously mentioned [39], followed by pretreatment with 10 mM citrate buffer (pH 6.0) for ten minutes at 98 °C to expose the antigen epitopes, and sealed with 5% goat serum. Next, the midgut tissue sections were cultured overnight with anti-PgCad1 antibody (1:350) at 4°C then incubated with goat anti-rabbit fluorescence antibody conjugated with tetraethyl rhodamine isothiocyanate (TRITC). After washing with PBS three times, blocking reagent was used to cover the slides and finally fluorescence analysis was conducted. We used a peptide containing 290 amino acid residues (from 22 to 311 amino acids) based on the cadherin protein PgCad1 encoded by the *s* allele (Figure S3) to generate the anti-PgCad1 antibody. The working concentration of the anti-PgCad1 antibody and the goat anti-rabbit fluorescence antibody was 4.4 µg/mL and 2.5 µg/mL, respectively.

### 4.10. Data Analysis

For diet bioassay data, we conducted probit regression by SPSS to analyze the LC_50_ values and 95% fiducial limits (FL), and slopes of the concentration–mortality lines and standard errors (SE). We also analyzed the cell toxicity data by probit regression to calculate the concentration of Cry1Ac causing swelling to half of the cells (EC_50_) and its 95% FL. In genetic linkage analysis, for the backcross offspring on a diet without Cry1Ac, we carried out a one-sample t-test to analyze if the observed proportion of individuals with genotype *r16r16* was significantly different from the 50% expected under random segregation. We conducted Fisher’s exact test to determine if the percentage of individuals with genotype *r16r16* survived on a diet with Cry1Ac was significantly different from that of a diet without Cry1Ac. For the boll bioassays, we carried out standard t-tests to analyze if the AQ65 resistant strain was significantly different from the APHIS-S susceptible strain in larval survival on both Bt and not-Bt cotton and relative survival. We conducted one-way ANOVA and Tukey’s HSD test to analyze if the development time and pupal weight differed significantly between AQ65 and APHIS-S on non-Bt cotton, and AQ65 on non-Bt cotton. For AQ65, we also carried out standard t-tests to determine if the development time, pupal weight, eggs laid per female, and hatching rate of eggs differed significantly between Bt and non-Bt cotton, and we used Fisher’s exact test to determine if significant differences occurred between Bt and non-Bt cotton in survival from neonate to adult and the percentage of female adults. 

## Figures and Tables

**Table 1:** Responses to Cry1Ac of pink bollworm larvae from a resistant strain (AQ65), a susceptible strain (APHIS-S), and their F_1_ progeny.

Strain or Cross	Slope (SE) ^a^	LC_50_ (95% FL) ^b^	RR ^c^
APHIS-S	3.78 (0.336)	0.097 (0.048–0.132)	
AQ65	1.79 (0.446)	29.5 (22.4–48.0)	300
AQ65♀ × APHIS-S♂	2.38 (0.203)	0.454 (0.494–0.623)	4.7
AQ65♂ × APHIS-S♀	2.94 (0.278)	0.559 (0.397–0.514)	5.8

^a^ Slope of the concentration–mortality line with its standard error in parentheses. ^b^ Concentration killing 50% with 95% fiducial limits in parentheses, in μg Cry1Ac per mL diet. ^c^ Resistance ratio, the LC_50_ for AQ65, AQ65♀ × APHIS-S♂ or AQ65♂ × APHIS-S♀ divided by the LC_50_ for APHIS-S.

**Table 2:** Time to pupation and pupal weight for pink bollworm on Bt and non-Bt cotton bolls.

Strain	Cotton Type	Number of Pupae	Time to Pupation (days)	Pupal wt. (mg)
APHIS-S	Non-Bt	70	15.0 ± 0.2 a	13.7 ± 0.4 a
AQ65	Non-Bt	85	17.1 ± 0.3 b	13.8 ± 0.4 a
AQ65	Bt	47	20.8 ± 0.4 c	11.6 ± 0.5 b

Values are means ± SE. Different lower case letters within columns indicate significant differences between treatments based on ANOVA followed by Tukey’s HSD.

**Table 3:** Life history traits of resistant pink bollworm strain AQ65 on Bt and non-Bt cotton bolls.

Trait	N		Bt	Non-Bt	Bt/non-Bt
	Bt	Non-Bt			
Neonate to adult survival	270	230	0.16	0.35	0.46
Proportion of females	43	80	0.37	0.45	0.82
Eggs per female	16	36	126 ± 26	171 ± 22	0.74
Hatch rate	1345	1607	0.82 ± 0.02	0.79 ± 0.03	1.04
Net reproductive rate ^a^			6.1	21.3	0.29

^a^ Net reproductive rate = neonate to adult survival x proportion of females x eggs per female x hatch rate [26].

## Supplementary Materials

The following are available online at https://www.mdpi.com/2072-6651/11/4/186/s1, Figure S1: Isolation of pink bollworm resistant strain AQ65; Figure S2: Alignment of the full-length cDNA of *s* and *r16* alleles; Figure S3: Predicted amino acid sequence of pink bollworm cadherin protein PgCad1 for alleles *s* (GenBank accession number MF276974) from susceptible strain APHIS-S, and *r16* (GenBank accession number KU254193) from resistant strain AQ65; Figure S4: Alignment of g and cDNA sequences of *r16* and *s* alleles; Figure S5: PCR detection of *PgCad1* genotype using primers in Table S1; Figure S6: Western blot of cadherin fusion proteins sPgCad1-GFP (lane 1) and r16PgCad1-GFP (lane 2) produced in Hi5 cells transfected with vectors containing the *s* and *r16* alleles, respectively. Table S1: Primers used for cloning and genotyping of *PgCad1*; Table S2: Responses to Cry2Ab of pink bollworm larvae from a resistant strain (AQ65) and a susceptible strain (APHIS-S); Table S3: Survival of AQ65 and APHIS-S larvae reared on Bt cotton and non-Bt cotton.
