# Supplementary material for: Pink Bollworm Resistance to Bt Toxin Cry1Ac Associated with an Insertion in Cadherin Exon 20
Source: Toxins (Basel). 2019 Mar 28;11(4):186. doi: 10.3390/toxins11040186 (PMC6521048; doi:10.3390/toxins11040186)
Supplement: Supplementary file 1 [file toxins-11-00186-s001.pdf]

Supplementary Materials: Pink Bollworm Resistance to Bt Toxin Cry1Ac Associated with an Insertion in Cadherin Exon 20

Ling Wang, Yuemin Ma, Xueqin Guo, Peng Wan, Kaiyu Liu, Shengbo Cong, Jintao Wang, Dong Xu, Yutao Xiao, Xianchun Li, Bruce E. Tabashnik and Kongming Wu

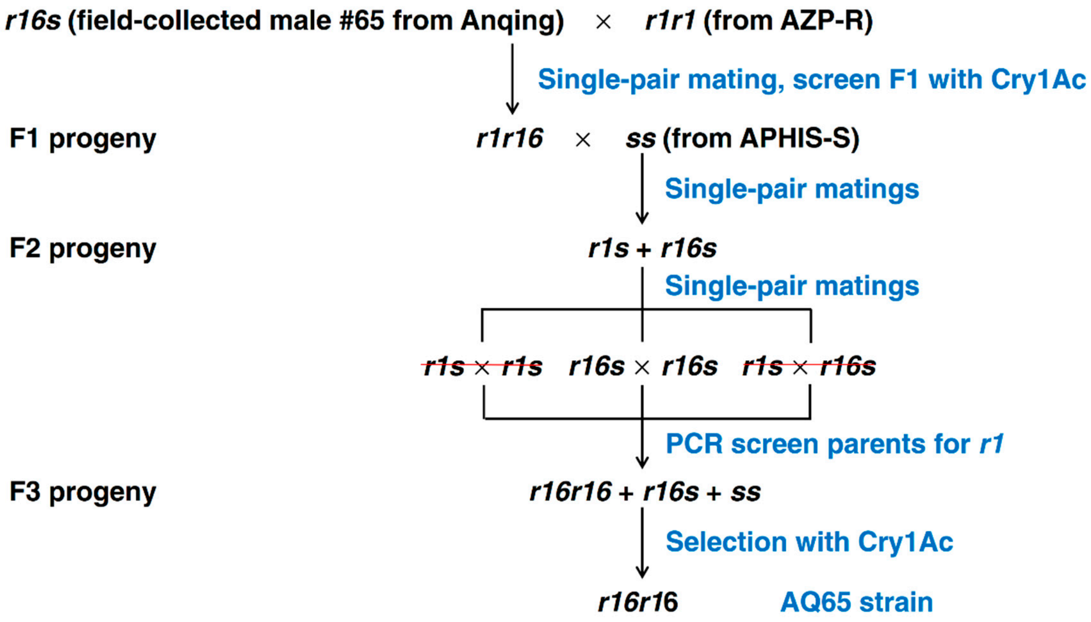

Figure S1. Isolation of pink bollworm resistant strain AQ65. The resistant strain AQ65 of pink bollworm was originated from a single-pair cross between a field-collected male (#65) from Anqing in Anhui province of the Yangtze River Valley and a female from a Cry1Ac-resistant strain AZP-R (cadherin genotype *r1r1*) from Arizona. Their F_1 offspring (family #65) were screened with a diagnostic concentration of Cry1Ac (10 μg Cry1Ac protoxin per mL diet). In view of the 40% survival of the F_1 progeny at the diagnostic concentration and recessive resistance to Cry1Ac of pink bollworm described before [1,2], it indicated that the male parent of family #65 carried only one recessive allele at *PgCad1* conferring Cry1Ac resistance. Sequencing of cDNA from the resistant F_1 offspring demonstrated that male #65 had only one transcript of cadherin allele, which we name *r16* (Genbank accession number KU254193) (Figures 1).

Survivors from family #65 are reared to adults, then each individual paired with a heterosexual adult from APHIS-S susceptible strain (cadherin genotype *ss*) from Arizona to generate resistant strain AQ65. PCR amplification was used to identify *r1* allele for the parents of all single pairs in F_2 generation [3], and only these single pairs that their parents did not carry *r1* allele (*r16s*) were retained, then the offspring larvae of these single pairs were selected on diet with the diagnostic concentration of Cry1Ac. The individuals survived on the diagnostic concentration were homozygotes *r16r16*, which were reared their offspring as strain AQ65, and feeding larvae of AQ65 with diet containing the diagnostic concentration of Cry1Ac every fifth generation to maintain the resistance to Cry1Ac.

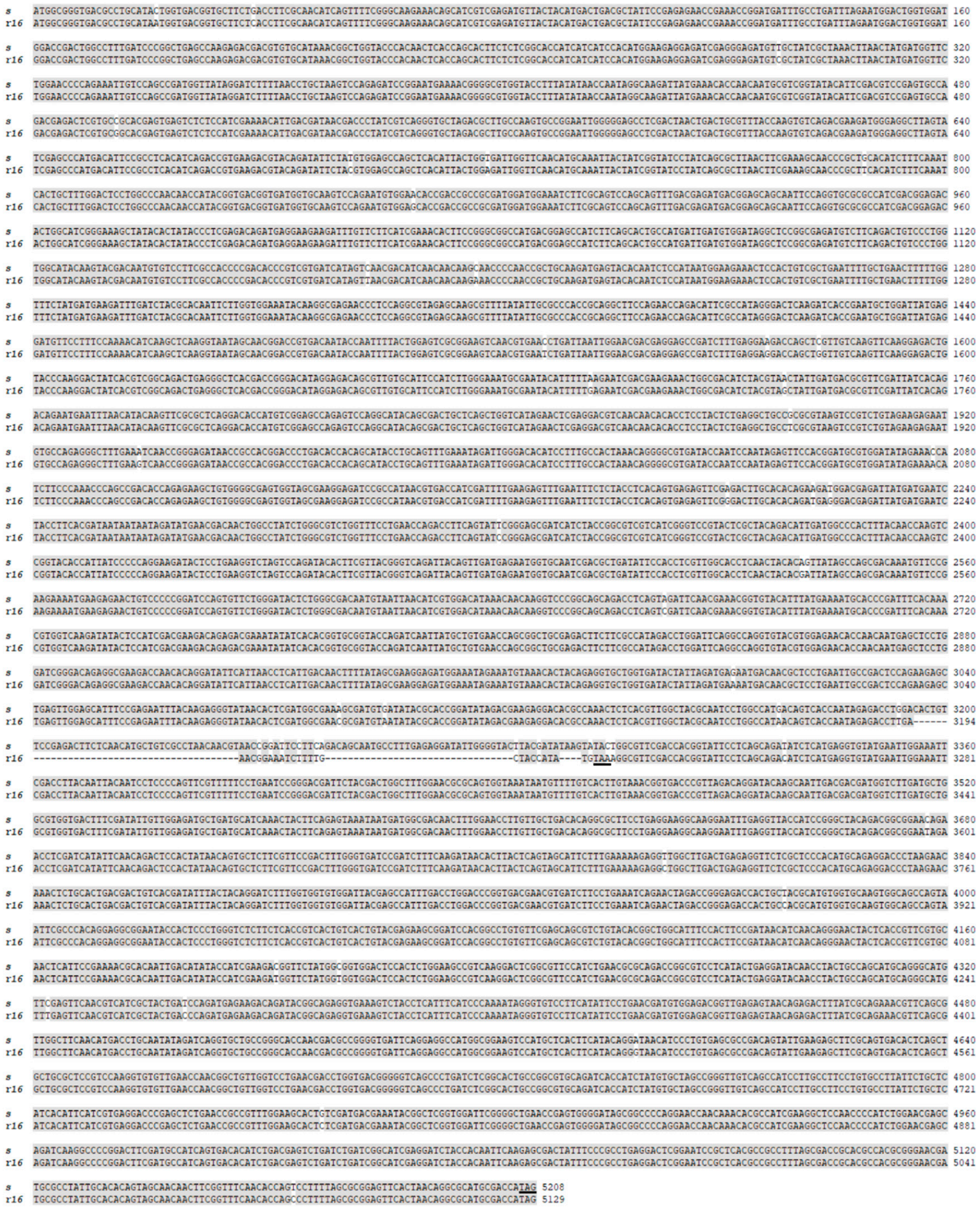

Figure S2. Alignment of the full-length cDNA of *s* and *r16* alleles. Underline letters TAA and TAG indicate termination codon of *r16* and *s*, respectively.

```
              SIG        <=
s    [MAGDACILVTVLLTFATSVFG]QETASSRCYYMTDAIPREPKPDDLPDLEWTGGWTDWPLIPAEPRDDVCINGWYPQLTSTSLGTIIIHMEEEIEGDVAIA 100
r16  -------M-------------------------------------------------------------------------------------------- 100
                               => CR1
s    KLNYDGSGTPEIVQPMVIGSFNLLSPEIRNENGAWYLYITNRQDYETPTMRRYTFDVRVPDETRAARVSLSIENIDDNDPIVRVLDACQVPELGEPRLTD 200
r16  ---------------------------------------------------------------------------------------------------- 200
         => CR2
s    CVYQVSDEDGRLSIEPMTFRLTSDREDVQIFYVEPAHITGDWFNMQITIGILSALNFESNPLHIFQITALDSWPNNHTVTVMVQVQNVEHRPPRWMEIFA 300
r16  ---------------------------------------------------------------------------------------------------- 300
                 => CR3
s    VQQFDEMTEQQFQVRAIDGDTGIGKAIHYTLETDEEEDLFFIETLPGGHDGAIFSTAMIDVDRLRRDVFRLSLVAYKYDNVSFATPTPVVIIVNDINNKQ 400
r16  ---------------------------------------------------------------------------------------------------K 400
        => CR4
s    PQPLQDEYTISIMEETPLSLNFAELFGFYDEDLIYAQFLVEIQGENPPGVEQAFYIAPTAGFQNQTFAIGTQDHRMLDYEDVPFQNIKLKVIATDRDNTN 500
r16  ---------------------------------------------------------------------------------------------------- 500
                          => CR5
s    FTGVAEVNVNLINWNDEEPIFEEDQLVVKFKETVPKDYHVGRLRAHDRDIGDSVVHSILGNANTFLRIDEETGDIYVTIDDAFDYHRQNEFNIQVRAQDT 600
r16  -----------------------------------------------------------------------------A---------------------- 600
                                          => CR6
s    MSEPESRHTATAQLVIELEDVNNTPPTLRLPRVSPSVEENVPEGFEINREITATDPDTTAYLQFEIDWDTSFATKQGRDTNPIEFHGCVDIETIFPNPAD 700
r16  ----------------------------------------------V---------------------------------------------N------- 700
                                                                            => CR7
s    TREAVGRVVAKEIRHNVTIDFEEFEFLYLTVRVRDLHTEDGRDYDESTFTIIIIDMNDNWPIWASGFLNQTFSIRERSSTGVVIGSVLATDIDGPLYNQV 800
r16  --------------------------------------DE------------------------------------------------------------ 800
                                                                                               => CR8
s    RYTIIPQEDTPEGLVQIHFVTGQITVDENGAIDADIPPRWHLNYTVIASDKCSEENEENCPPDPVFWDTLGDNVINIVDINNKVPAADLSRFNETVYIYE 900
r16  ---------------------------------------------I------------------------------------------------------ 900

s    NAPDFTNVVKIYSIDEDRDEIYHTVRYQINYAVNQRLRDFFAIDLDSGQVYVENTNNELLDRDRGEDQHRIFINLIDNFYSEGDGNRNVNTTEVLVILLD 1000
r16  ---------------------------------------------------------------------------------------------------- 1000
           => CR9
s    ENDNAPELPTPEELSWSISENLQEGITLDGESDVIYAPDIDEEDTPNSHVGYAILAMTVTNRDLDTVPRLLNMLSPNNVTGFLQTAMPLRGYWGTYDISI 1100
r16  -------------------------------R------------------------I-------E-EIFCYH-*                           1074
                                                           => CR10
s    LAFDHGIPQQISHEVYELEIRPYNYNPPQFVFPESGTILRLALERAVVNNVLSLVNGDPLDRIQAIDDDGLDAGVVTFDIVGDADASNYFRVNNDGDNFG 1200
r16
                                                                              => CR11
s    TLLLTQALPEEGKEFEVTIRATDGGTEPRSYSTDSTITVLFVPTLGDPIFQDNTYSVAFFEKEVGLTERFSLPHAEDPKNKLCTDDCHDIYYRIFGGVDY 1300
r16
                                                                   => CR12
s    EPFDLDPVTNVIFLKSELDRETTATHVVQVAASNSPTGGGIPLPGSLLTVTVTVREADPRPVFEQRLYTAGISTSDNINRELLTVRATHSENAQLTYTIE 1400
r16
                                                                              => MPR
s    DGSMAVDSTLEAVKDSAFHLNAQTGVLILRIQPTASMQGMFEFNVIATDPDEKTDTAEVKVYLISSQNRVSFIFLNDVETVESNRDFIAETFSVGFNMTC 1500
r16
                                                                                     => TM
s    NIDQVLPGTNDAGVIQEAMAEVHAHFIQDNIPVSADSIEELRSDTQLLRSVQGVLNQRLLVLNDLVTGVSPDLGTAGVQITIYVLAGLSAILAFLCLILL 1600
r16
        => CYT
s    ITFIVRTRALNRRLEALSMTKYGSVDSGLNRVGIAAPGTNKHAIEGSNPIWNEQIKAPDFDAISDTSDESDLIGIEDLPQFKSDYFPPEDSESAHAAFSD 1700
r16

s    RTPRGNDAPIAHSSNNFGFNTSPFSAEFTNRRMRP 1735
r16
```

Figure S3. Predicted amino acid sequence of pink bollworm cadherin protein PgCad1 for alleles *s* (GenBank accession number MF276974) from susceptible strain APHIS-S; and *r16* (GenBank accession number KU254193) from resistant strain AQ65. The *s* was sequenced in 2015 from the subset of APHIS-S reared in China [4]. Shown are the signal sequence (SIG), cadherin repeats (CR1-CR12), membrane-proximal region (MPR), transmembrane region (TM) and cytoplasmic region (CYT). The short dashes for *r16* indicate the sequence is the same as for the *s* allele. The asterisk at position 1074 in *r16* indicates that, because of the premature stop codon (Figure S2), the *r16* allele encodes only 1073 amino acids.

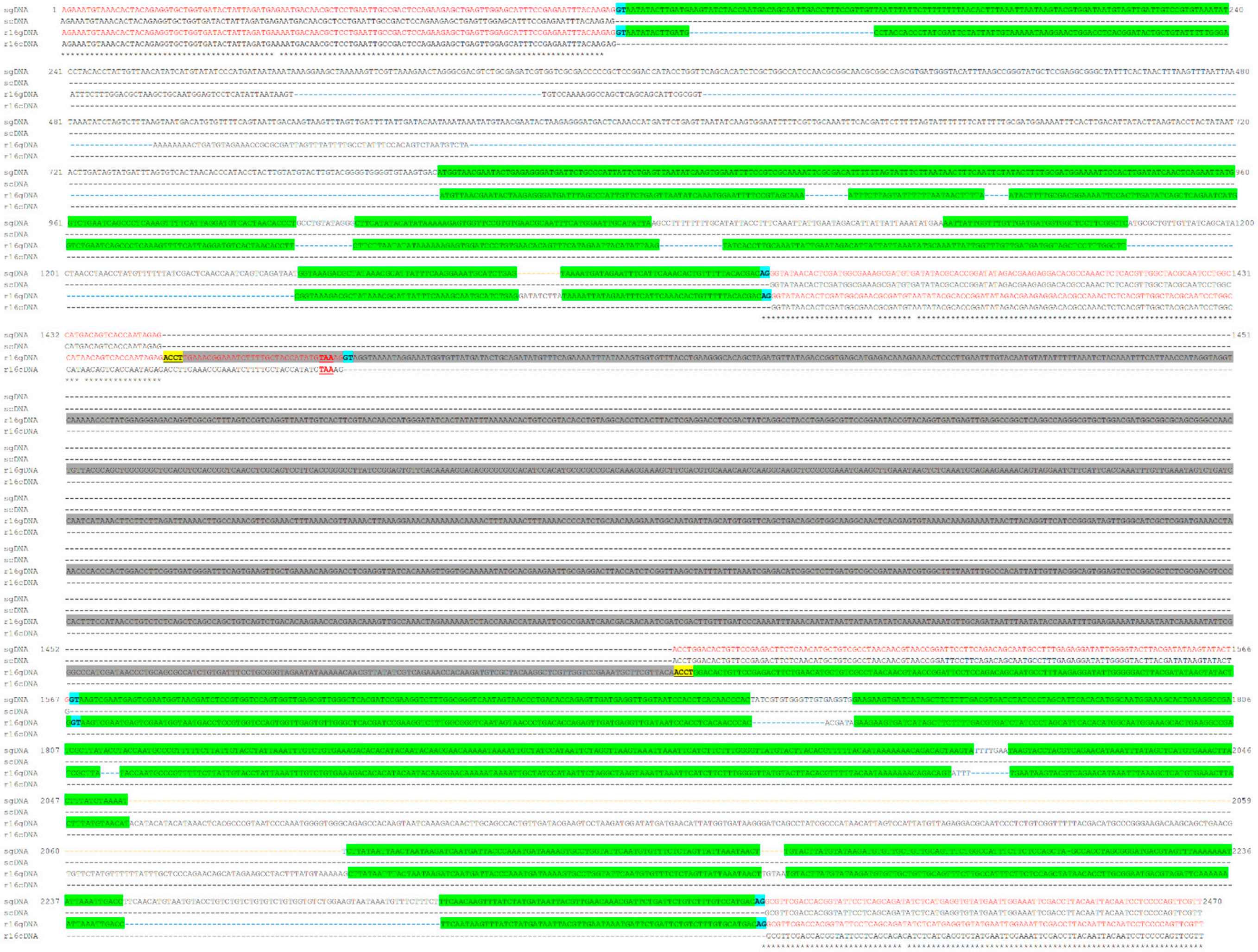

Figure S4. Alignment of g and cDNA sequences of *r16* and *s* alleles. The red letters indicate exon sequences. The green background represents the consistent sequence of introns in *r16* and *s*. The blue background GT/AG indicate splicing site. The yellow background ACCT indicate target site duplicates (TSDs). The grey background indicates the inserted transposon sequence. The red and underline letters TAA indicate premature stop codon in *r16*.

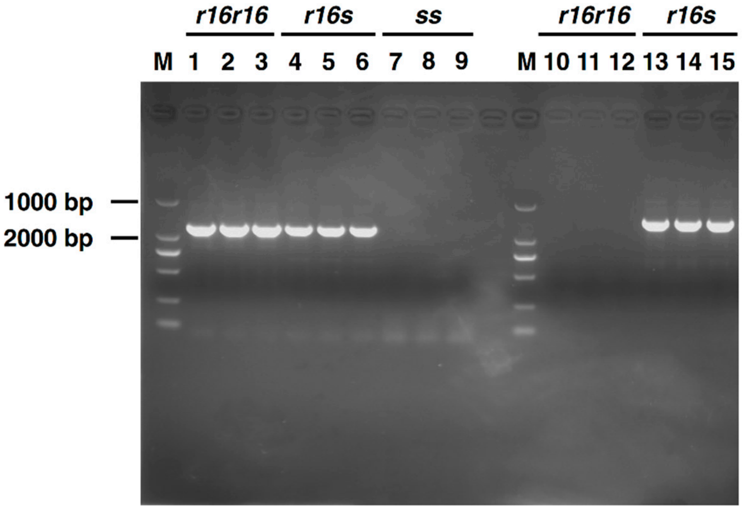

Figure S5. PCR detection of *PgCad1* genotype using primers in Table S1. Primers for *r16* (*r16*allF and *r16*R) generate a single band of 1211 bp in *r16r16* from AQ65 (lanes 1–3) and in *r16s* (F_1 progeny of APHIS-S × AQ65; lanes 4–6); and no band in *ss* from APHIS-S (lanes 7–9). Primers for *s* (*r16*allF and not*r16*R) generate no band from *r16r16* (lanes 10–12) and a single band of 1431 bp from *r16s* (lanes 13–15).

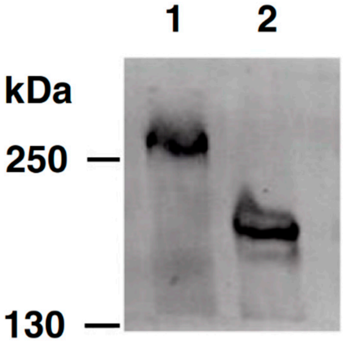

Figure S6. Western blot of cadherin fusion proteins sPgCad1-GFP (lane 1) and r16PgCad1-GFP (lane 2) produced in Hi5 cells transfected with vectors containing the *s* and *r16* alleles, respectively.

References

1. Morin, S.; Biggs, R.W.; Sisterson, M.S.; Shriver, L.; Ellers-Kirk, C.; Higginson, D.; Holley, D.; Gahan, L.J.; Heckel, D.G.; Carrière, Y.; et al. Three cadherin alleles associated with resistance to *Bacillus thuringiensis* in pink bollworm. *Proc. Natl. Acad. Sci. USA.* **2003**, *100*, 5004–5009.
2. Tabashnik, B.E.; Morin, S.; Unnithan, G.C.; Yelich, A.J.; Ellers-Kirk, C.; Harpold, V.S.; Sisterson, M.S.; Ellsworth, P.C.; Dennehy, T.J.; Antilla, L.; et al. Sustained susceptibility of pink bollworm to Bt cotton in the United States. *GM Crops & Food.* **2012**, *3*, 194–200.
3. Morin, S.; Henderson, S.; Fabrick, J.A.; Carriere, Y.; Dennehy, T.J.; Brown, J.K.; Tabashnik, B.E. DNA-based detection of Bt resistance alleles in pink bollworm. *Insect Biochem. Mol. Biol.* **2004**, *34*, 1225–1233.
4. Wang, L.; Ma, Y.; Wan, P.; Liu, K.; Xiao, Y.; Wang, J.; Cong, S.; Xu, D.; Wu, K.; Fabrick, J.A.; et al. Resistance to *Bacillus thuringiensis* linked with a cadherin transmembrane mutation affecting cellular trafficking in pink bollworm from China. *Insect Biochem. Mol. Biol.* **2018**, *94*, 28–35.

Table S1. Primers used for cloning and genotyping of *PgCad1*.

Name[a]	Primer sequence (5′–3′)	Template	Strain	Size (bp)
F1	CATACTGGTGACGGTGCTTCT	cDNA	APHIS-S	2384
R1	GGACTTGGTTGTAAAGTGGGC		AQ65	2384
F2	GACCTTCAGTATTCGGGAGCG	cDNA	APHIS-S	2890
R2	CATGCGCCTGTTAGTGAACTC		AQ65	2811
gF65	AGAAATGTAAACACTACAGAGGTGC	gDNA	APHIS-S	2470
gR65	AACGAACTGGGGAGGATTGTAATTG		AQ65	3664
*r16*allF	GATGAAAATGACAACGCTCCTG	gDNA	APHIS-S	None
*r16*R	CGAGTAAGTGAGGTGCCTACAG		AQ65	1211
*r16*allF	GATGAAAATGACAACGCTCCTG	gDNA	APHIS-S	1431
not*r16*R	CTCGGAACAGTGTCCAGGTCT		AQ65	None
PgCADF	CCGGAATTCGCCACCATGGCGGGTGA<br>CGCCTGCAT	cDNA	APHIS-S	5205
PgCADR	TCCCCGCGGACCGCCTCCGCCACCG<br>CCCATATGGTAGCAAAAGATTTCCGT	cDNA	AQ65	3219

[a] F indicates forward and R reverse.

Table S2. Responses to Cry2Ab of pink bollworm larvae from a resistant strain (AQ65) and a susceptible strain (APHIS-S).

Strain.	Slope (SE)[a]	LC_{50} (95% FL)[b]	RR[c]
APHIS-S	2.69 (0.339)	0.157 (0.125–0.188)	
AQ65	3.42 (0.402)	0.408 (0.350–0.467)	2.60

[a]Slope of the concentration-mortality line with its standard error in parentheses. [b]Concentration killing 50% with 95% fiducial limits in parentheses, in μg Cry2Ab per ml diet. [c]Resistance ratio, the LC_{50} for AQ65 divided by the LC_{50} for APHIS-S.

Table S3. Genetic linkage between resistance to Cry1Ac and cadherin gene *PgCad1*.

	Larvae with *r16r16* (%)	
Backcross family	Control diet	Cry1Ac diet
1	43	100
2	53	100
3	55	100
4	43	100
5	46	100
Mean	48	100

We used PCR (Fig S3) to determine the genotype for a total of 250 larvae: 147 on control diet (30, 30, 29, 30 and 28 larvae from backcross families 1–5, respectively) and 103 on diet treated with the diagnostic concentration of Cry1Ac (20, 20, 21, 22 and 20 larvae from backcross families 1–5, respectively).

Table S4. Survival of AQ65 and APHIS-S larvae reared on Bt cotton and non-Bt cotton.

Insect strain	Cotton type	Bolls	Entry holes per boll	Survivors/ boll	Survival(%) [a]	Relative survival (%) [b]
AQ65	Bt	48	5.6 (0.3)	1.1 (0.1)	19.4 (1.6)	50.6 (2.7)
APHIS-S	Bt	35	5.6 (0.1)	0.0 (0.0)	0.0 (0.0)	0.0 (0.0)
AQ65	Non-Bt	44	5.2 (0.3)	2.0 (0.1)	38.3 (2.8)	
APHIS-S	Non-Bt	43	5.5 (0.2)	1.7 (0.1)	31.1 (0.9)	

Values are means with their standard errors in parentheses. [a]Larvae surviving per boll divided by entry holes per boll multiplied by 100%. [b]Survival on Bt cotton divided by survival on non-Bt cotton.
